# Impact of orphan drugs on Latvian budget

**DOI:** 10.1186/s13023-016-0434-y

**Published:** 2016-05-11

**Authors:** Konstantins Logviss, Dainis Krievins, Santa Purvina

**Affiliations:** Department of Pharmacology, Riga Stradins University, 13 Pilsonu Street, Riga, LV-1002 Latvia; Department of Education and Science, Pauls Stradins Clinical University Hospital, 13 Pilsonu Street, Riga, LV-1002 Latvia; University of Latvia, 19 Raina Boulevard, Riga, LV-1586 Latvia

**Keywords:** Orphan drugs, Budget impact, Expenditure, Reimbursement, Latvia

## Abstract

**Background:**

Number of orphan medicinal products on the market and number of rare disease patients, taking these usually expensive products, are increasing. As a result, budget impact of orphan drugs is growing. This factor, along with the cost-effectiveness of orphan drugs, is often considered in the reimbursement decisions, directly affecting accessibility of rare disease therapies. The current study aims to assess the budget impact of orphan drugs in Latvia.

**Methods:**

Our study covered a 5-year period, from 2010 to 2014. Impact of orphan drugs on Latvian budget was estimated from the National Health Service’s perspective. It was calculated in absolute values and relative to total pharmaceutical market and total drug reimbursement budget. A literature review was performed for comparison with other European countries.

**Results:**

Orphan drug annual expenditure ranged between EUR 2.065 and 3.065 million, with total 5-year expenditure EUR 12.467 million. It constituted, on average, 0.84 % of total pharmaceutical market and 2.14 % of total drug reimbursement budget, respectively. Average annual per patient expenditures varied widely, from EUR 1 534 to EUR 580 952. The most costly treatment was enzyme replacement therapy (Elaprase) for MPS II. Glivec had the highest share (34 %) of the total orphan drug expenditure. Oncological drugs represented more than a half of the total orphan drug expenditure, followed by drugs for metabolic and endocrine conditions and medicines for cardiopulmonary diseases. Three indications: Ph+ CML, MPS II, and PAH accounted for nearly 90 % of the total orphan drug expenditure.

**Conclusions:**

Budget impact of orphan drugs in Latvia is very small. It increased slightly over a period of five years, due to the slight increase in the number of patients and the number of orphan drugs reimbursed. Current Latvian drug reimbursement system is not sufficient for most orphan drugs.

## Background

Orphan drugs are medicinal products intended for the treatment, diagnosis or prevention of life-threatening or seriously debilitating rare conditions affecting less than one person in 2 000 individuals across the European Union [1]. Most of them are indicated for treatment of oncological conditions [2–5], followed by metabolic and endocrine diseases (including lysosomal storage diseases) and cardiovascular disorders (particularly pulmonary arterial hypertension). By the end of 2014, there were more than a thousand positive opinions on orphan designation, but less than 80 orphan drugs with active marketing authorization in the EU [6, 7].

Orphan medicinal products are often highly expensive [8]. Factors, such as costs of research and development, marketing exclusivity, lack of alternative therapies, disease severity, and small market size can affect orphan drug prices. Moreover, orphan drugs for treatment of diseases with lower prevalence generally have higher costs than drugs indicated to treat more common conditions [3, 9]. Especially drugs for ultra-orphan diseases [10], with a prevalence of less than 1 per 50 000 persons, are highly expensive. In fact, orphan designated drugs tend to have higher prices than non-designated drugs for rare diseases [11]. Standard cost-effectiveness criteria are often not applicable to orphan drugs [4, 8], considering the high costs of these medicines and often modest health gains.

Budget impact of orphan drugs is growing, which puts pressure on decision makers. Given current fiscal constraints and financial uncertainty around orphan drugs, health authorities are increasingly concerned about the growth in orphan drug expenditure and its impact on their limited budgets. On the one hand, budget impact for an individual orphan drug is usually small [12, 13], due to the limited numbers of patients. The majority of orphan drugs have relatively low sales [13], except few high-cost orphan drugs. Though, budget impact for orphan drugs altogether might be considerable [12, 14]. As a result, the number of studies conducted in this field at the European level is increasing. An early European study showed that orphan medicinal products accounted for 0.7 % and 1 % of total drug budgets in France and the Netherlands in 2004 [9], and it was anticipated to increase to 6–8 % by 2010. Another study focused on five European countries with the highest drug expenditures, and found that the average overall impact of orphan drugs was 1.7 % of total drug spending in 2007 [3]. The impact of orphan drugs was predicted to increase from 3.3 % of the total European pharmaceutical market in 2010 to a peak of 4.6 % in 2016 [14]. In Europe, total budget impact of ultra-orphan drugs for non-oncological diseases was estimated to be EUR 20 625 million over 10 years (2012–2021) [15]. In Belgium, orphan drugs represented 1.9 % (EUR 66 million) of total drug expenditure in 2008 [16, 17], and it was estimated to grow to about 4 % (EUR 162 million) in 2013. The share of total pharmaceutical expenditure spent on orphan drugs in the Netherlands almost quadrupled, from 1.1 % in 2006 to 4.2 % in 2012 (from EUR 61.2 to 260.4 million) [12]. In 2012, budget impact of orphan medicinal products represented 2.5 % of total pharmaceutical market in Sweden and 3.1 % in France [13]. And it was forecasted to grow to 4.1 % in Sweden and 4.9 % in France by 2020. Finally, in Bulgaria, which was a pioneering country in Eastern Europe in adopting a national plan and implementing registers for rare diseases, expenditure for outpatient rare disease therapies was around 8 % of the total National Health Insurance Fund’s expenditure for medications in 2012–2014 (about EUR 32 million in 2013) [5].

Orphan drug availability, accessibility, pricing and reimbursement policies differ between European countries [4, 18, 19]. Some countries consider the budget impact and cost-effectiveness of orphan drugs in their reimbursement decisions. For example, in France, Italy, the Netherlands, the UK, and Serbia reimbursement is based on both, cost-effectiveness and budget impact. In contrast, Belgium and Bulgaria do not consider the cost-effectiveness, while the budget impact analysis is not required in Sweden.

In Latvia, there is no specific policy in place for the pricing and reimbursement of orphan medicinal products [20, 21]. Cost-effectiveness and expected budget impact are evaluated for each medicine before inclusion in the reimbursement list. Drug prices are compared with prices in selected European countries. Orphan drugs can be provided through the three main mechanisms. Firstly, the reimbursement system covers medicinal products included in the national reimbursement drug list. Medicines can also be reimbursed within the framework of individual reimbursement system, with annual limit of EUR 14 229 (previously LVL 10 000) per patient. Additionally, orphan drugs for children are provided through the state funded program “Medicinal treatment for children with rare diseases”.

It has been shown that a low gross domestic product (GDP) value and availability of a formal health technology assessment (HTA) organization have negative influence on orphan drug market uptake [22]. Budget impact analyses conducted so far focused predominantly on the old EU countries with a high GDP (markets with high drug expenditures) or Europe as a whole [3, 9, 12–17]. In contrast, Latvian study could provide an insight on the situation in a small Eastern European country with a low GDP. The current study aims to assess the budget impact of orphan drugs in Latvia and compare it with other European countries.

## Methods

Our study covered a 5-year period, from 2010 to 2014. Orphan drugs were defined as the medicinal products with European marketing authorization and European orphan designation granted by the European Medicines Agency (EMA) and active during the studied period. European Community register of designated orphan medicinal products and the EMA database of rare disease designations were used to identify orphan drugs authorized in the EU. Some drugs, that were originally designated orphan medicines, are no longer considered orphan drugs in Europe. These products were withdrawn from the European Community register of designated orphan medicinal products, either at the end of the period of market exclusivity or on the request of the sponsor. We included such drugs in the study until the end of the year when the last orphan indication of the product was withdrawn, i.e. the last year these drugs were formally considered orphan medicines in Europe. For instance, Sutent was withdrawn from the European Community register in 2008, and was, therefore, out of the scope of the current study, which covered the period of 2010–2014.

Impact of orphan drugs on Latvian budget was calculated from the National Health Service’s (NHS) perspective. A particular orphan drug can have multiple indications, orphan and non-orphan. For such drugs, only expenditures related to orphan indications were taken into account. For assessment of orphan drug reimbursement, including the reimbursement lists and the individual reimbursement, we used the NHS annual reports on the use of funds for reimbursement of outpatient drugs and medical devices. Children’s Clinical University Hospital (CCUH) purchase procedure reports on the “Medicinal treatment for children with rare diseases” program (financed by the NHS) were analyzed to assess orphan drugs provided through this pathway. A particular orphan medicinal product can be provided through multiple reimbursement mechanisms. For such products, double counting was excluded. Total drug reimbursement budget (orphan and non-orphan products) was calculated as a sum of funds covering the reimbursement lists, individual reimbursement and the CCUH program. This information was available from the NHS annual public reports. For the information on total pharmaceutical market (total turnover of medicines) we used “Statistics on Medicines Consumption” annually published by the State Agency of Medicines of Latvia. Budget impact of orphan drugs was calculated by dividing the expenditures covering orphan drugs by the total pharmaceutical market and the total drug reimbursement budget, respectively.

Euro (EUR) was introduced in Latvia on 1st January 2014, thus no currency conversion was required for this year. For the period 2010–2013, we used the official exchange rate defined by the Bank of Latvia for the national currency (Latvian lat – LVL). Starting from 2005, the Bank of Latvia set a fixed exchange rate 1 EUR = 0.7028 LVL, which was actual until the end of 2013. When the literature review was performed, for comparison with other countries, we used xe.com EUR exchange rates, if orphan drug expenditures were expressed in other currencies, e.g. British pounds (GBP).

Annual expenditure per patient was calculated by dividing the annual expenditure covering orphan drug reimbursement by the number of patients receiving these medications. For the individual reimbursement and the CCUH program, the number of patients receiving particular drugs was known from the NHS annual reports on the use of funds for reimbursement of outpatient drugs and medical devices and the NHS annual public reports, respectively. For drugs included in one of the reimbursement lists, the number of patients was estimated by using the EMA approved summary of product characteristics (SPC) and the number of drug packages reimbursed by the NHS. The SPC was used to identify the recommended maintenance daily dose used for drug's main indication in adults. This dose was further converted to the number of pharmaceutical forms (e.g. tablets or capsules). Then, the content of a single package was divided by the number of pharmaceutical forms required per day, to find the duration of treatment (number of days) covered by one package. It was further calculated how many packages are required for one year treatment period. Number of patients receiving a particular drug was estimated by dividing the total number of drug packages reimbursed by the NHS by the number of packages required for one patient per year.

We performed a literature review to compare the budget impact of orphan drugs in Latvia with other European countries. The budget impact of orphan drugs was expressed in absolute figures (million EUR) and relative to the total pharmaceutical market. If a study reported only the budget impact of orphan drugs as a percentage of the total pharmaceutical market, the absolute figures were calculated taking into account the numbers representing the total pharmaceutical market, as reported in the study. If multiple studies were available for a country, the most recent study was selected. If a study reported actual (observed) data and data forecasted for the future, the observed data for the latest year were preferred. The World Bank’s data on the population and GDP (PPP) per capita were used for each country for the year of interest. We also identified the number of orphan drugs with active marketing authorizations in the EU and converted the orphan drug expenditure into expenditure per 100 000 inhabitants.

## Results

Twenty one different orphan drugs were reimbursed through the three reimbursement pathways during the period covered by the study (Table 1). The number of orphan medicines reimbursed per year increased slightly, from 11 drugs in 2010 to 15 drugs in 2014. Four drugs were provided through multiple reimbursement mechanisms: Sprycel and Wilzin were provided individually prior to inclusion in the reimbursement list; Glivec was simultaneously reimbursed individually and through the reimbursement list; Cystadane was provided through the individual reimbursement and the CCUH program. Nplate and Mozobil were included in the reimbursement list in 2014 and 2015, respectively, however so far these products were reimbursed individually. Aldurazyme and Sutent were reimbursed after the loss of orphan drug status in the EU, and were, therefore, excluded from the study.

**Table 1:** Orphan drugs reimbursed in Latvia

Reimbursement Lists				
Trade Name	Active Substance	Orphan Indication	Inclusion Date	Reimbursement List
Glivec*	Imatinib	Ph+ CML; Ph+ ALL; MDS/MPD; GIST; DFSP; HES and CEL	April 2013	List A (previously List C)
Nplate	Romiplostim	Idiopathic thrombocytopenic purpura (ITP)	March 2014	List B
Wilzin*	Zinc	Wilson’s disease	June 2014	
Sutent*	Sunitinib	GIST	December 2014	
Sprycel	Dasatinib	Ph+ CML; Ph+ ALL	October 2010	List C
Tasigna	Nilotinib	Ph+ CML	December 2010	
Mozobil	Plerixafor	HSCT in patients with lymphoma and multiple myeloma	January 2015	
CCUH Program				
Trade Name	Active Substance	Orphan Indication		
Elaprase	Idursulfase	Hunter syndrome (Mucopolysaccharidosis II – MPS II)		
Myozyme	Alglucosidase alpha	Pompe disease		
Aldurazyme*	Laronidase	Mucopolysaccharidosis I (MPS I)		
Kuvan	Sapropterin	Hyperphenylalaninaemia (HPA) in patients with phenylketonuria (PKU) or tetrahydrobiopterin (BH4) deficiency		
Cystadane	Betaine	Homocystinuria		
Increlex	Mecasermin	Primary insulin-like growth factor 1 deficiency (primary IGFD)		
Votubia	Everolimus	Renal angiomyolipoma and subependymal giant cell astrocytoma associated with tuberous sclerosis complex (TSC)		
Individual Reimbursement				
Trade Name	Active Substance	Orphan Indication		
Revatio	Sildenafil	Pulmonary arterial hypertension (PAH)		
Volibris	Ambrisentan	PAH		
Tracleer*	Bosentan	PAH; systemic sclerosis		
Nexavar	Sorafenib	Hepatocellular carcinoma; renal cell carcinoma; differentiated (papillary/follicular) thyroid carcinoma		
Atriance	Nelarabine	T-ALL and T-LBL		
Sutent*	Sunitinib	GIST		
Glivec*	Imatinib	Ph+ CML; Ph+ ALL; MDS/MPD; GIST; DFSP; HES and CEL		
Sprycel	Dasatinib	Ph+ CML; Ph+ ALL		
Mozobil	Plerixafor	HSCT in patients with lymphoma and multiple myeloma		
Arzerra	Ofatumumab	Chronic lymphocytic leukaemia (CLL)		
Nplate	Romiplostim	ITP		
Revolade*	Eltrombopag	ITP		
Exjade	Deferasirox	Chronic iron overload due to blood transfusions in patients with beta thalassaemia major, other anaemias, and non-transfusion-dependent thalassaemia syndromes		
Wilzin*	Zinc	Wilson’s disease		
Cystadane	Betaine	Homocystinuria		
Diacomit	Stiripentol	Dravet’s syndrome (Severe myoclonic epilepsy in infancy – SMEI)		
Drugs withdrawn from the European Community register of designated orphan medicinal products				
Trade Name	Active Substance	Withdrawal Date	Reason of Withdrawal	
Aldurazyme	Laronidase	June 2013	End of the period of market exclusivity	
Wilzin	Zinc	October 2014		
Revolade	Eltrombopag	January 2012	Request of the sponsor	
Sutent	Sunitinib	July 2008		
Glivec	Imatinib	November 2011	End of the period of market exclusivity (for Ph+ CML)	
		April 2012	Request of the sponsor (for other indications)	
Tracleer	Bosentan	May 2012	End of the period of market exclusivity (for PAH)	
		April 2014	Request of the sponsor (for systemic sclerosis)	

*Drugs withdrawn from the European Community register of designated orphan medicinal products

**Fig. 1:**
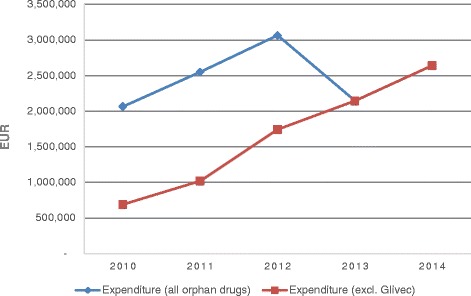
Orphan drug expenditure

Orphan drug annual expenditure ranged between EUR 2.065 and 3.065 million, with total 5-year expenditure EUR 12.467 million (Table 2). It constituted, on average, 0.84 % of the total pharmaceutical market annually, with a maximum 1.04 % seen in 2012, followed by a minimum 0.70 % in 2013. These peak and bottom values can be explained by the fact that Glivec was withdrawn from the European Community register of designated orphan medicinal products in 2012, and was no longer considered orphan medicine in the EU. Additionally, after the patent expiration in 2013, imatinib generics became available and practically replaced the brand drug from Latvian drug reimbursement system. Orphan drugs represented, on average, 2.14 % of the total drug reimbursement annual budget, with maximal (2.62 %) and minimal (1.83 %) values also observed in 2012 and 2013. If Glivec was excluded from the study, the orphan drug expenditure would increase constantly, from EUR 0.692 million to EUR 2.642 million (Fig. 1). It corresponds to more than a threefold increase, from 0.25 % to 0.84 % of the total pharmaceutical market within 5 years.

**Table 2:** Budget impact of orphan drugs

Trade name	Active substance	Expenditure (EUR)						Share of total expenditure	Reimbursement category
		2010	2011	2012	2013	2014	Total		
Atriance	Nelarabine	24 773					24 773	0.20 %	Individual reimbursement
Nexavar	Sorafenib	16 418	12 040	23 806	17 786	14 229	84 278	0.68 %	
Revatio	Sildenafil	104 742	147 370	219 252	340 536	480 685	1 292 585	10.37 %	
Volibris	Ambrisentan	52 172	87 744	142 289	128 058	163 633	573 897	4.60 %	
Exjade	Deferasirox	5 726				7 329	13 055	0.10 %	
Sprycel	Dasatinib	60 450					60 450	0.48 %	
Wilzin	Zinc	1 317	169				1 486	0.01 %	
Cystadane	Betaine	1 038	3 142	2 094	4 327	6 560	17 161	0.14 %	
Diacomit	Stiripentol	6 696	8 940	14 354	11 424	16 947	58 360	0.47 %	
Glivec	Imatinib		47 731	68 608			116 340	0.93 %	
Arzerra	Ofatumumab		27 830				27 830	0.22 %	
Mozobil	Plerixafor		12 796	25 592			38 389	0.31 %	
Nplate	Romiplostim		13 687				13 687	0.11 %	
Revolade	Eltrombopag		3 790				3 790	0.03 %	
Tracleer	Bosentan					12 697	12 697	0.10 %	
Glivec	Imatinib	1 373 374	1 481 110	1 252 280			4 106 764	32.94 %	List A (previously List C)
Sprycel	Dasatinib		202 997	296 703	412 538	524 119	1 436 357	11.52 %	List C
Tasigna	Nilotinib			149 561	275 615	285 964	711 140	5.70 %	
Wilzin	Zinc					1 534	1 534	0.01 %	List B
Elaprase	Idursulfase	418 275	501 228	596 232	681 408	707 619	2 904 762	23.30 %	CCUH program
Kuvan	Sapropterin			173 374	173 374	213 828	560 575	4.50 %	
Cystadane	Betaine			6 265	6 265	6 416	18 945	0.15 %	
Increlex	Mecasermin			94 257	93 557	93 820	281 634	2.26 %	
Myozyme	Alglucosidase alpha					71 548	71 548	0.57 %	
Votubia	Everolimus					34 802	34 802	0.28 %	
All Orphan Drugs		2 064 981	2 550 574	3 064 669	2 144 887	2 641 727	12 466 838		
Total Pharmaceutical Market		276 690 000	290 190 000	295 480 000	307 590 000	316 040 000	Average		
Share of Total Pharmaceutical Market		0.75 %	0.88 %	1.04 %	0.70 %	0.84 %	0.84 %		
Total Drug Reimbursement Budget		105 911 722	118 204 402	117 007 689	117 384 880	122 326 421	Average		
Share of Total Drug Reimbursement		1.95 %	2.16 %	2.62 %	1.83 %	2.16 %	2.14 %

**Fig. 2:**
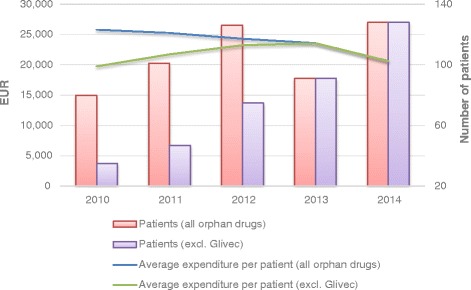
Number of patients and average annual expenditure per patient

In a 5-year period, number of patients receiving orphan drugs increased by 60 %, from 80 to 128 patients. It changed in a similar manner as the orphan drug expenditure, depending on Glivec exclusion (Fig. 2). Until 2012, Glivec had the highest annual number of patients, varying between 40 % and 56 % of all patients in 2010–2012, whereas Revatio had the highest growth in the number of patients, with more than a fourfold increase within 5 years (from 18 to 77 patients, i.e. 60 % of all patients in 2014). The average overall annual expenditure per patient decreased by 20 %, from EUR 25 812 to EUR 20 638.

**Fig. 3:**
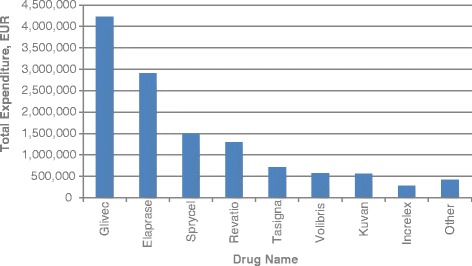
Orphan drugs with the highest total expenditure

Average annual per patient expenditures varied widely, from EUR 1 534 for Wilzin to EUR 580 952 for Elaprase, and averaged at EUR 23 701 (Table 3). Annual budget for Elaprase grew constantly, with a peak per patient expenditure reaching EUR 707 619 in 2014. More than a half of the total orphan drug expenditure within 5 years was the expenditure related to two medications, Glivec (33.9 %) and Elaprase (23.3 %) (Fig. 3). Moreover, considering the fact that Glivec was included in the study until the end of 2012, these two products generated 86.8 %, 79.6 %, and 62.6 % in three consecutive years 2010–2012.

**Table 3:** Average annual expenditure per patient

Trade Name	Active Substance	Average annual expenditure per patient (EUR)
Glivec	Imatinib	30 420; 7 756^a^
Sprycel	Dasatinib	49 530; 60 450^a^
Tasigna	Nilotinib	47 409
Wilzin	Zinc	1 534; 495^a^
Mozobil	Plerixafor	12 796
Nplate	Romiplostim	13 687
Elaprase	Idursulfase	580 952
Myozyme	Alglucosidase alfa	71 548
Increlex	Mecasermin	40 233
Kuvan	Sapropterin	43 121
Votubia	Everolimus	34 802
Cystadane	Betaine	6 315; 3 432^a^
Revatio	Sildenafil	6 185
Volibris	Ambrisentan	10 434
Tracleer	Bosentan	12 697
Diacomit	Stiripentol	7 295
Arzerra	Ofatumumab	13 915
Atriance	Nelarabine	12 386
Nexavar	Sorafenib	10 535
Exjade	Deferasirox	6 527
Revolade	Eltrombopag	3 790

^a^Indicates the individual reimbursement, if a drug was provided through multiple reimbursement mechanisms

**Fig. 4:**
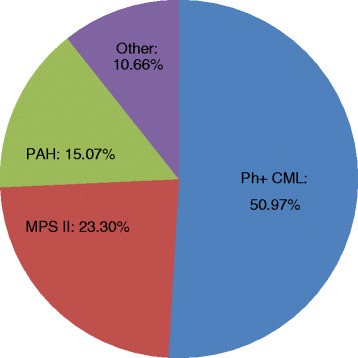
Orphan drug indications with the highest total expenditure

Oncology drugs represented 52.99 % of the total orphan drug expenditure, followed by drugs for metabolic and endocrine conditions (30.94 %) and medicines for cardiopulmonary diseases (15.07 %). More specifically, Ph+ CML treatment agents (Glivec, Sprycel, and Tasigna) generated 50.97 % of the total orphan drug expenditure, followed by Elaprase for MPS II (23.30 %) and drugs for PAH (Revatio, Volibris, and Tracleer), with 15.07 % (Fig. 4). Although, these drugs were provided through different reimbursement mechanisms: the total expenditure covering orphan drugs provided through the reimbursement lists was almost exclusively represented by the agents for Ph+ CML (99.98 %), whereas Elaprase and drugs for PAH amounted to 75.01 % of the CCUH program and 80.35 % of the individual reimbursement orphan drug expenditures, respectively.

Budget impact of orphan drugs as a proportion of the total pharmaceutical market is 3–5 times smaller in Latvia than in other recently studied (in 2012) markets (Table 4). Latvian population is 5–40 times smaller and GDP (PPP) per capita is 1.5–2 times smaller than in other countries. Consequently, the orphan drug expenditure per 100 000 inhabitants is 2–12 times smaller in Latvia. This difference is remarkable, considering the time lag between the studies and the different number of orphan drugs authorized in the EU (44 orphan drugs in 2007 vs. 78 in 2014). For example, more than a million EUR was spent per 100 000 inhabitants in three countries in 2012, when the number of orphan drugs on the market was closer to the number seen in 2014.

**Table 4:** Budget impact of orphan drugs in European countries

Country	Budget impact of OD relative to the total drug expenditure	Budget impact of OD (million EUR)	Year	Number of OD with active MA in the EU^a^	Population (million)^a^ [26]	GDP (PPP) per capita^a^ [31]	OD expenditure per 100 000 inhabitants (EUR)^a^
UK	1.0 % [3]	162.0	2007	44	61.32	37 507	264 188
Italy	1.5 % [3]	235.5	2007	44	58.44	33 731	402 977
Spain	2.0 % [3]	256.0	2007	44	45.23	32 807	565 996
Germany	2.1 % [3]	525.0	2007	44	82.27	36 782	638 143
Belgium	1.9 % [16, 17]	66.2	2008	49	10.71	37 847	618 114
Netherlands	4.2 % [12]	260.4	2012	64	16.75	46 379	1 554 627
France	3.1 % [13]	1054.0	2012	64	65.64	37 256	1 605 728
Sweden	2.5 % [13]	107.2	2012	64	9.52	43 869	1 126 050
Latvia	0.84 %	2.64	2014	78	1.99	22 873	132 663

*OD* orphan drugs, *MA* marketing authorization, *GDP (PPP) per capita* gross domestic product per capita based on purchasing power parity

^a^In the year of interest

A study by Picavet et al. included Latvia in the European analysis of orphan drug market uptake [22]. Latvia was clustered with Hungary and Poland, as countries with a low GDP and a formal HTA organization. This cluster had the lowest orphan drug market volumes and sales. Moreover, in Latvia, only EUR 8 000 was spent per 100 000 inhabitants on orphan medicines, compared to approximately EUR 560 000 in France. The share of orphan drug sales relative to the total drug market sales varied from 0.07 % in Latvia to 1.90 % in Estonia. These results are critical for Latvia. However, there are some details that should be clarified here. Only 17 orphan drugs were included in the analysis, out of which only five drugs were launched in Latvia. Moreover, orphan drugs which generated the highest expenditures in our study (Glivec, Elaprase, and Revatio) were not included. Therefore, the real market uptake of all orphan drugs authorized in the EU is much higher, although it relates to all European markets, rather than specifically to Latvia. Our study found that the orphan drug expenditure constituted, on average, 0.84 % of the total pharmaceutical market in Latvia. It increased very slightly over a period of five years, remaining under the 1 % threshold, due to the slight increase in the number of patients and the number of orphan drugs reimbursed, whereas the average annual expenditure per patient decreased. In contrast, in the Netherlands, budget impact of orphan drugs increased almost fourfold over a period of six years [12], while both, the number of patients and the number of orphan drugs, almost quadrupled.

Orphan drug expenditures in Latvia are characterized by extremely small numbers, considering a trend of orphan drug budget impact to increase, due to the growing number of orphan medicinal products on the market and the growing number of patients taking these, usually expensive, products. Similar figures were reported only in the very first budget impact studies, when the number of orphan drugs on the European market was small. Thus, orphan medicinal products accounted for 0.7 % and 1 % of total drug budgets in France and the Netherlands in 2004 [9], when only 15 orphan drugs were authorized in the EU. More recent analyses showed greater impact of orphan drugs on total pharmaceutical market. For instance, the share of total pharmaceutical expenditure spent on orphan drugs in the Netherlands increased markedly, from 1.1 % in 2006 to 4.2 % in 2012 [12]. A study of five European countries, with the highest drug expenditure, found that the average overall impact of orphan drugs was 1.7 % of total drug spending in 2007 [3]. In Belgium, orphan drugs accounted for 1.9 % of total drug expenditure in 2008 [16, 17], and it was estimated to grow to about 4 % in 2013. It should be noted that less than 50 orphan drugs had European marketing authorizations at the time of these two studies, in 2007–2008. Later, Schey et al. predicted the impact of orphan drugs to increase from 3.3 % of the total European pharmaceutical market in 2010 to a peak of 4.6 % in 2016 [14]. Finally, in 2012, budget impact of orphan medicinal products accounted for 2.5 % of total pharmaceutical market in Sweden and 3.1 % in France [13]. These budget impact analyses focused predominantly on the old EU countries with a high GDP. The current study demonstrated that budget impact of orphan drugs in Latvia, as a small Eastern European country with a low GDP and, hence, healthcare budget constraints, is considerably lower. It is, however, complicated to compare the budget impacts of orphan medicines in different countries due to a lack of country-specific epidemiological data for orphan diseases, differences in pricing and reimbursement systems, and time lag between the studies.

Different studies used different approaches and sources of information for the analyses, such as the estimates of budget impact submitted by pharmaceutical companies in the original reimbursement files (or revision files submitted later, when more recent information is available), data published by the HTA organizations, NHS or other payers, and IMS (Intercontinental Medical Statistics) Health data. IMS Health database is widely used to evaluate and compare drug markets. Though, it has been pointed out that there are some differences in data quality between countries [22]. Moreover, real utilization of medicines may differ from that reflected in sales data. There are both, retrospective and forecasting, types of budget impact analyses. Actual data are used as a basis for forecasts, while forecasting nature of the analyses usually leaves much uncertainty, depending on multiple variables, such as number of orphan designations and marketing authorizations, drug costs, number of patients, reimbursement decisions, availability of therapeutic alternatives, and competition after expiration of marketing exclusivity and patent protection. Budget impact analyses have been criticized for their simplicity [4, 17, 23]. The analyses are generally limited to evaluating the impact of drug costs, rather than total treatment costs. If an orphan drug has multiple indications, budget impact across all indications is often not considered, likewise the potential savings, if there is alternative treatment available.

## Discussion

### Reimbursement system

Availability and accessibility of orphan drugs in a particular country depend on multiple factors, such as marketing strategy of pharmaceutical companies, market attractiveness, pricing and reimbursement policies. Orphan drugs can be reimbursed through the three main mechanisms in Latvia: the reimbursement list, the individual reimbursement, and the CCUH program “Medicinal treatment for children with rare diseases”. The national reimbursement list consists of three parts [20, 21]: List A covers therapeutically equivalent drugs (generics); List B consists of medicines without therapeutic equivalents; and List C contains expensive drugs, for which the annual costs exceed EUR 4 269 (previously LVL 3 000) per patient, and the manufacturer is obliged to cover treatment expenses (not less than 10 %) for a certain number of patients. This provision is an important tool to manage the costs of expensive medicines, although it is not intended specifically for orphan drugs. For example, Glivec was additionally covered by the company for five patients in 2010 and 2011 [24, 25]. Orphan drugs are usually included in the List C. Starting from 2014, some orphan drugs are also included in the List B (Table 1). The individual reimbursement can be provided only if a disease is not included in the reimbursement list, or the disease is included in the list, but there are no drugs included in the reimbursement list for treatment of this condition. Not more than 2 % of the national drug reimbursement budget is intended to the individual reimbursement, with a limitation up to EUR 14 229 (previously LVL 10 000) per patient per year.

Orphan drugs reimbursed in Latvia can be divided in two groups. Drugs provided through the reimbursement lists and the CCUH program can generally be considered fully accessible to patients, whereas drugs provided through the individual reimbursement are frequently only partially accessible, considering the annual limit of EUR 14 229 per patient. This threshold is too low. Only Revatio, Diacomit, Cystadane, Wilzin, and Mozobil can be fully provided within this limit. Other orphan drugs should be additionally covered by the manufacturers, charities or patients themselves.

### Market size

Number of orphan medicines reimbursed per year through the three reimbursement pathways increased slightly, reaching 15 drugs in 2014. It is less than 20 % out of 78 orphan drugs with active marketing authorizations in the EU in the same year. The remaining drugs are practically inaccessible to rare disease patients. Decisions to launch the product on the market and to apply for the reimbursement are taken by the manufacturer. Market size plays a crucial role in these decisions. The absolute number of rare disease patients treated in Latvia is very low. Some orphan drugs (including Elaprase) were reimbursed for a single patient. There might be no diagnosed patients eligible for the treatment with a particular orphan drug. In 2014, a total of 128 patients received orphan drugs in Latvia. In contrast, in the Netherlands, Glivec alone was provided to 1 485 patients in 2012 [12]. The low number of patients along with the fiscal constraints make Latvian market less attractive for the manufacturers of orphan drugs.

Orphan drugs are generally less reimbursed in new EU Member States [9], whose health care budgets are considerably lower than those of older Member States. Bulgarian study reported similar findings, where over two-thirds of orphan drugs were not reimbursed in 2014 [5]. Authors compared this number with other EU Member States, where about 80 % of orphan medicinal products are incorporated in the healthcare systems. They also pointed out that time delay from the EU marketing authorization to the positive reimbursement decision is much longer in Bulgaria than in other countries. Bulgaria is bigger country than Latvia, with a population 7.2 million vs. 2.0 million in Latvia [26], and consequently more rare disease patients. According to the Eurordis survey, especially smaller countries suffer from longer delay in availability of orphan medicines [27]. Differences in the annual per patient costs for a given orphan drug can reach 70 % between the EU countries [9]. Besides, orphan drug prices are higher in the smaller of new EU Member States, such as the Baltics, than in the bigger states, such as Poland or the Czech Republic.

### Therapeutic areas of orphan drugs

Oncological drugs represented more than a half of the total orphan drug expenditure, followed by the drugs for metabolic and endocrine conditions and the medicines for cardiopulmonary diseases. Those are generally the main therapeutic areas of orphan drugs [2–5]. In 2010, oncological and haematological disorders accounted for 57 % of the total orphan drug costs in Europe [14]. Within these therapeutic areas, there are some indications, for which either multiple orphan drugs or highly expensive orphan drugs are available. Thereby, nearly 90 % of the total orphan drug expenditure in our study covered only three indications: Ph+ CML, MPS II, and PAH. One of such orphan drugs for Ph+ CML treatment, Glivec, is a blockbuster anticancer drug with multiple orphan indications. It generated 34 % of the total orphan drug expenditure within 5 years. Similar results were reported in other studies. The majority of orphan drugs have relatively low sales [13], except few high-cost orphan drugs. For instance, the total sales of Glivec reached EUR 679 million in the five biggest European countries in 2007 [3]. It was more than 40 % of the total orphan drug expenditure. Moreover, if the expenditures relating to three drugs (including imatinib) with the highest sales were excluded from the study, budget impact of the remaining orphan medicines would be more than halved. In our study, it would be enough to exclude just two medications (Glivec and Elaprase) to reach the same result. In a Dutch study, Glivec also had the highest cumulative budget impact (EUR 251.2 million) [12]. It accounted for 34 % of the total orphan drug expenditure between 2000 and 2012 in Sweden, and 27 % in France [13].

### Intellectual property

Loss of intellectual property, such as expiration of marketing exclusivity and patent protection, can greatly affect drug prices and result in an increased competition. As reported by Onakpoya et al., for orphan drugs, where generic alternatives were available, the branded products were from 1.4 to 82 000 times more expensive [10]. However, it is not clear yet whether the orphan drug market is attractive enough for generic companies to enter the field of rare diseases. Orphan drug market has distinctive features, characterized primarily by specific European regulation, small number of patients, and high drug prices. In addition, orphan medicines have remarkably higher proportion of large-molecule than small-molecule agents [2], compared to non-orphan drugs. Since the biologicals are currently less subjected to generic (biosimilar) competition than the small molecules, they can maintain high economic value even after the patent expiration. The current study demonstrated that generic companies may have a big interest in some orphan drugs. Starting from May 2013, Glivec was moved to the reimbursement List A, because generic drugs became available, that changed prescribing and reimbursement criteria for imatinib. In fact, cheaper imatinib generics practically replaced the brand drug from the reimbursement system. In 2014, the reimbursement expenditure covering Glivec was only EUR 2 904, compared to the annual expenditure varying between EUR 1.321 and 1.529 million in 2010–2012. However, Glivec should not be considered as a model for all orphan drugs, since it is a small molecule, used for multiple indications, and known for a long time as a classical blockbuster orphan drug. Not all orphan medicines are expected to cause such interest from the generic companies. It should be noted that orphan drugs were excluded from the analysis when the period of market exclusivity ended. It is likely that these drugs will still have a budgetary impact, as patients will continue using them. However, these products were removed from the Community register of orphan medicinal products and are no longer considered orphan medicines in Europe.

### Enzyme replacement therapy

Annual per patient costs can vary broadly between different orphan drugs: EUR 1 534–580 952 (current study); EUR 6 000–300 000 [9]; EUR 331–337 501 [3]; EUR 1 251–407 631 [14]; GBP 726–378 000 [10]. In the present study, the two most expensive drugs, on the annual per patient basis, were Elaprase and Myozyme. Both medicines were provided through the CCUH program, as enzyme replacement therapy (ERT) for MPS II and Pompe disease. The program provided ERT also for Gaucher disease (Cerezyme) and MPS I (Aldurazyme), however these products are not considered orphan drugs in Europe. In fact, if Cerezyme and Aldurazyme were included in the study, they would be among the most expensive medicines, with the average annual per patient expenditures EUR 213 716 and EUR 157 248, respectively, and more than EUR 1 million of the total expenditure in 5 years.

ERT for Gaucher disease was the most costly per patient therapy in Israel [28]. To decrease the costs authors recommended to apply criteria of disease severity, use low-dose regimen or even “drug vacations”. In Bulgaria, MPS and glycogen storage disease (conditions treated with ERT) were the two rare diseases with the highest costs per patient [5]. Elaprase and Naglazyme had the highest estimated annual costs among the inpatient orphan drugs in the Netherlands [29], whereas Myozyme had the highest budget impact. However, it appears that ERT is not the most expensive treatment worldwide. Soliris (eculizumab), for the treatment of paroxysmal nocturnal haemoglobinuria, was mentioned as the most expensive drug in the world [2], with annual cost around USD 500 000 in 2010. The latest price record was set by Glybera (alipogene tiparvovec) [30], the first gene therapy drug approved by the EMA for lipoprotein lipase deficiency in 2012, with a cost over EUR 1 million per patient. Both drugs are designated orphan medicinal products in the EU.

### Future budget impact of orphan drugs

Orphan drug expenditure grew faster (with annual growth rates 20–25 % in the years not affected by the change in the status of Glivec) than the total pharmaceutical market (annual growth rates 2–5 %) and the total drug reimbursement budget (Table 5). The only negative growth (−30 %) was observed in 2012–2013, that was caused by the change in the status of Glivec. Based on the observed trends, it is likely that the budget impact of orphan drugs in Latvia will follow the general European tendencies and will continue to grow in the future, both in absolute numbers and relative to the total pharmaceutical market. This assumption is strengthened by the fact that the number of orphan drugs will only increase in the future, both at European level (14 new orphan drugs were approved by the EMA in 2015) and at Latvian national level (3 orphan drugs were included in the reimbursement list in 2014–2015). Other studies have shown that the budget impact of orphan drugs in European countries is increasing, however the growth rates are decreasing over time [12–14], due to expiration of patents and marketing exclusivity of existing orphan drugs. It is, therefore, likely that the budget impact of orphan drugs in Latvia will remain sustainable and relatively small in the long run. Although, it should be pointed out that currently available data is too limited to create a detailed and well validated model for the reliable forecast of the future budget impact of orphan drugs in Latvia. Further research is needed to identify the trends of orphan drugs, including the detailed information on the availability and accessibility of orphan drugs (including the time lag between the orphan drug marketing approval in the EU and the inclusion in the reimbursement system in Latvia), the potential patient population, and the prices of orphan drugs.

**Table 5:** Annual growth rates

Expenditure	2010–2011	2011–2012	2012–2013	2013–2014
Orphan drugs (all)	23.52 %	20.16 %	−30.01 %	23.16 %
Orphan drugs (excl. Glivec)	47.73 %	70.67 %	23.00 %	23.16 %
Total pharmaceutical market	4.88 %	1.82 %	4.10 %	2.75 %
Total drug reimbursement budget	11.61 %	−1.01 %	0.32 %	4.21 %

### Limitations

A limitation of the current study is, in fact, that the annual per patient expenditures were estimated from the payer’s (NHS) perspective only. For drugs provided within the individual reimbursement system the actual drug costs may be much higher, considering the limit of EUR 14 229 per patient per year covered by the NHS. If the drug cost exceeds this limit, the rest of expenses should be covered by the manufacturers, charities or patients. Information concerning the expenses not covered by the NHS is not publicly available, although it should not have direct impact on Latvian healthcare budget. Thus, for orphan drugs reimbursed individually the annual per patient expenditures may be considered as the actual drug costs, only if the above mentioned limit was not exceeded, i.e. for Revatio, Diacomit, Cystadane, Wilzin, and Mozobil.

Another limitation of our study can be found in the different approach for estimating the number of patients receiving particular drugs. For the individual reimbursement and the CCUH program this number was known from the NHS reports, while for orphan drugs included in one of the reimbursement lists the number of drug packages reimbursed by the NHS was known instead. To estimate the number of patients receiving such drugs we considered the recommended maintenance daily doses used for the main indications in adults. Therefore, the estimated number of patients for Sprycel and Tasigna (indicated in adults only) could be closer to the actual number of patients than for Glivec and Wilzin, which are indicated in both, adult and pediatric patients. It should be noted that not all patients are treated for a whole year and with the recommended maintenance doses. Additionally, for drugs used for Ph+ CML treatment the main indication was considered Ph+ CML in chronic phase, rather than accelerated or blast phases.

## Conclusions

Latvia is in a position of “a small market within the small market” or “ultra-small market” for orphan drugs, considering the small population, low GDP, healthcare budget constraints, and imperfections in drug reimbursement system. Currently, budget impact of orphan drugs in Latvia is very small compared to other European countries. Orphan drug expenditure is expected to increase in the future, as more orphan drugs will become available, both at European and Latvian level. However, in the long run, the growth rate of the orphan drug expenditure is expected to diminish and level off, as patents and marketing exclusivity of existing orphan drugs will expire. It is, therefore, likely that the budget impact of orphan drugs in Latvia will remain sustainable and relatively small.

Patient access to rare disease therapies in Latvia needs to be improved considering the disease severity and unmet medical needs, while the orphan drug expenditure should be efficiently managed. This is challenging but achievable through enhanced cooperation between all stakeholders and implementation of different reimbursement mechanisms, such as various types of risk-sharing agreements and conditional reimbursement programs, which link the reimbursement to health and economic outcomes. These mechanisms can be combined with rare disease registers and post-marketing surveillance programs that capture clinical and economic data and monitor orphan drug uptake. In this context, international cooperation and European collaboration are of crucial importance.

## Abbreviations

BH4: tetrahydrobiopterin
CCUH: Children’s Clinical University Hospital
CEL: chronic eosinophilic leukaemia
CLL: chronic lymphocytic leukaemia
DFSP: dermatofibrosarcoma protuberans
EMA: European Medicines Agency
ERT: enzyme replacement therapy
EU: European Union
EUR: euro
GBP: British pound sterling
GDP: gross domestic product
GIST: gastrointestinal stromal tumours
HCV: hepatitis c virus
HES: hypereosinophilic syndrome
HSCT: haematopoietic stem cell transplantation
HTA: health technology assessment
IGFD: insulin-like growth factor 1 deficiency
IMS: Intercontinental Medical Statistics
ITP: idiopathic thrombocytopenic purpura
LVL: Latvian lat
MDS/MPD: myelodysplastic/myeloproliferative diseases
MPS: mucopolysaccharidosis
MRCC: metastatic renal cell carcinoma
NHS: National Health Service
PAH: pulmonary arterial hypertension
Ph+ ALL: Philadelphia chromosome positive acute lymphoblastic leukaemia
Ph+ CML: Philadelphia chromosome positive chronic myeloid leukaemia
PKU: phenylketonuria
pNET: pancreatic neuroendocrine tumours
SMEI: severe myoclonic epilepsy in infancy
SPC: summary of product characteristics
T-ALL: T-cell acute lymphoblastic leukaemia
T-LBL: T-cell lymphoblastic lymphoma
TSC: tuberous sclerosis complex
USD: United States dollar
